# Carotid-Cavernous Fistula in a Patient With Unilateral Periorbital Swelling and Proptosis Initially Attributed to Possible Immunoglobulin G4-Related (IgG4-Related) Disease

**DOI:** 10.7759/cureus.39608

**Published:** 2023-05-28

**Authors:** Elizabeth Peterson, Artem Minalyan, Christina Downey

**Affiliations:** 1 Medicine, Loma Linda University School of Medicine, Loma Linda, USA; 2 Medicine/Rheumatology, Loma Linda University Health, Loma Linda, USA

**Keywords:** igg4-related disease, chemosis, eye pain, proptosis, periorbital swelling, carotid-cavernous fistula

## Abstract

The differential diagnosis for proptosis and periorbital swelling is broad and includes infectious, malignant, vascular, and rheumatologic etiologies. In this study, we report a case of carotid-cavernous fistula as the cause of acute-onset unilateral proptosis and periorbital swelling of the right eye in a 44-year-old female patient whose symptoms were initially attributed to possible immunoglobulin G4-related disease (IgG4-RD). The patient initially received antibiotics for presumed cellulitis and steroid treatment for a possible autoimmune cause, however; her autoimmune work-up was negative. Radiologic imaging later confirmed that she had a direct spontaneous carotid-cavernous fistula. She experienced significant improvement in her symptoms and vision after embolization treatment. Due to the risk that a carotid-cavernous fistula will progress quickly and cause neurological damage, this is a key diagnosis that should not be missed in patients with acute-onset periorbital and visual symptoms. Rheumatologists should include this condition in the differential for any patient who presents with periorbital swelling and vision disturbances.

## Introduction

The differential diagnosis for unilateral and isolated proptosis and periorbital swelling is broad and includes infectious, malignant, vascular, and rheumatologic etiologies. Of the rheumatologic conditions that affect periorbital structures, the recently defined immunoglobulin G4-related disease (IgG4-RD) presents a diagnostic challenge given its varying degree of multiorgan involvement and the lack of definitive tests for diagnosis [1]. While diagnosis often requires histopathologic analysis as well as the fulfillment of specific criteria, the potential for this condition to inflict long-term organ damage and its general responsiveness to immunosuppression justify assessing for this condition relatively early in the absence of other obvious diagnoses [1,2]. However, because of the non-specific presentation of IgG4-related disease, many other pathologies overlap in clinical presentation, and these must remain on the differential during the autoimmune work-up to avoid misdiagnosis and ensure timely and effective treatment for patients. In this study, we report a case of carotid-cavernous fistula diagnosed in a patient who was being worked up for possible IgG4-related disease.

## Case presentation

The patient is a 44-year-old female with a past medical history of migraines who was referred to our institution from the emergency department of a regional hospital with a chief complaint of swelling of the right eye. Over the course of one day, she had developed periorbital swelling, right eye proptosis, and right eye irritation that had escalated to a throbbing 7/10 pain by the time she presented to our emergency department. The regional hospital that referred the patient had diagnosed her with right periseptal cellulitis and possible early right orbital cellulitis based on CT imaging. She had received one dose of intravenous antibiotics and was discharged on clindamycin and cefalexin. She presented to our institution for an ophthalmology evaluation the next day with worsening swelling. Associated symptoms included diplopia that preceded the swelling, as well as blurry vision, headache, nausea, intermittent diplopia, and ptosis that worsened at the end of the day. She did not have any recent eye trauma, fevers, chills, weight loss, shortness of breath, chest pain, abdominal pain, joint pain or swelling, or rash.

Initial ophthalmology examination showed right periorbital swelling and proptosis (Hertel exophthalmometry of 20 mm in the right eye and 18 mm in the left) without redness or warmth around the right eye. She did have chemosis inferiorly in the right eye. She had diminished visual acuity in the right eye (20/40, 20/30 pinhole) compared to the left (20/20). Her pupils were equal and reactive without an afferent pupillary defect. Her extraocular muscle movements were full in the left eye, but she had decreased infraduction and adduction in the right eye. Confrontational visual fields were full in both eyes. She was found to have ocular hypertension in both eyes and was started on acetazolamide, brimonidine eyedrops, dorzolamide/timolol eyedrops, and latanoprost eyedrops. However, the pressure in the right eye remained persistently elevated (28 mmHg, with normal intraocular pressure ranging from 12 mmHg to 21 mmHg) during this therapy. The ophthalmology team recommended that she complete her previously prescribed per oral (PO) antibiotics, start prednisone at 1 mg/kg daily, and consider an orbital biopsy if there was no improvement in the swelling with steroids. A CT scan of the orbits with contrast at this time showed asymmetric enlargement of the right extraocular muscles and superior ophthalmic vein and the suggestion of asymmetric fullness of the right cavernous sinus. The chest X-ray was unremarkable.

**Figure 1:**
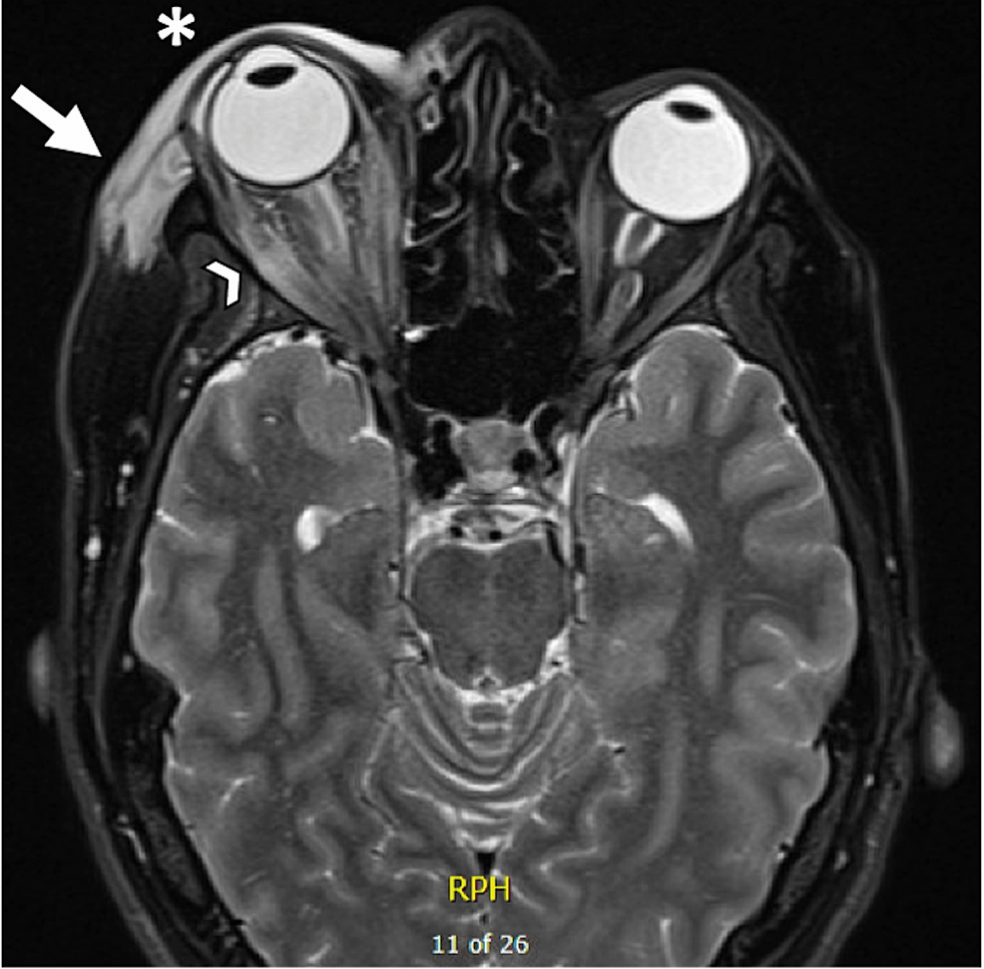
MRI orbit with contrast: T-2 weighted image showing increased enhancement and edema of the right extraocular muscles and retrobulbar fat (see arrowhead) and right periorbital soft tissues (see arrow) with associated proptosis (see asterisk). MRI: magnetic resonance imaging

In the following two days, the patient’s eye pain improved, but she had no improvement in the periorbital swelling. An MRI brain venography with and without contrast showed normal caliber and flow signals in the superior sagittal sinus, torcular herophili, transverse sinuses, sigmoid sinuses, and internal jugular veins, with no filling defects visible. MRI of the brain and orbits with contrast showed soft tissue swelling in the right orbit without lacrimal gland involvement (Figure 1).

With no improvement in the periorbital swelling seen by the third day of hospitalization, ophthalmology recommended starting pulse-dosed steroids (methylprednisolone IV 1 g for two days), which resulted in some improvement of the patient’s periorbital edema but unchanged chemosis.

After the patient’s two-day IV methylprednisolone course had finished, she was started on oral methylprednisolone, 64 mg (0.85 mg/kg) daily. Lab workup by this time included erythrocyte sedimentation rate, C-reactive protein, and complete blood count (CBC), which were unremarkable apart from microcytic anemia with elevated red blood cell distribution width. Chemistries were notable for a slightly decreased sodium of 133 mEq/L and bicarbonate of 19 mEq/L. Pro-B-type natriuretic peptide (Pro-BNP), hemoglobin A1C, and urinalysis were all unremarkable.

Other workups included a flow cytometry lymphoma profile and labs for thyroid stimulating immunoglobulins, anti-muscle-specific kinase antibodies, acetylcholine receptor binding antibodies, antithyroid peroxidase antibodies, anti-angiotensin-I-converting enzyme antibodies, syphilis, an anti-neutrophilic cytoplasmic antibody panel, lactate dehydrogenase activity, serum protein electrophoresis, an antinuclear antibody panel, vasculitis antibodies, thyrotropin receptor antibodies, and thyroglobulin antibodies, which were all unremarkable. A fractionated IgG showed minimally decreased IgG (762 mg/dL), normal IgG4, and elevated IgG3 (144 mg/dL) concentrations.

**Figure 2:**
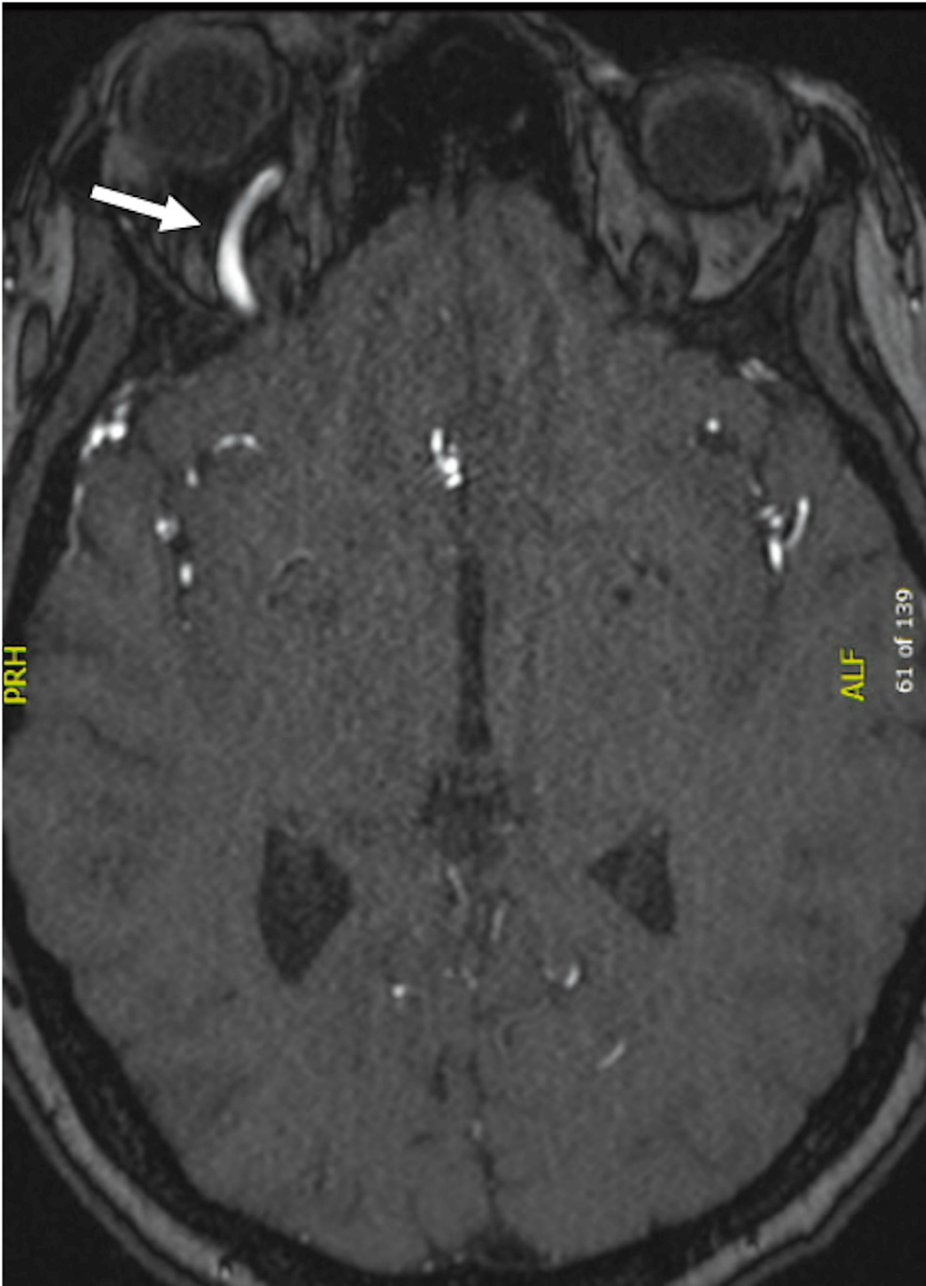
MRI brain angiography with contrast: TOF3D image showing increased flow-related signal in the dilated right superior ophthalmic vein (see arrow) as well as in the right cavernous and petrosal sinuses, suspicious for a carotid-cavernous fistula. MRI: magnetic resonance imaging

Given the patient’s partial response to steroids in the setting of a largely unremarkable lab work-up, rheumatology was consulted for evaluation for possible IgG4-related disease, sarcoidosis, or an idiopathic orbital inflammatory syndrome, and treatment recommendations for a steroid-sparing therapy. By this time, the patient had received a total of two doses of prednisone 80 mg, two days of IV methylprednisolone 1 g daily, one day of methylprednisolone 64 mg, and was receiving fluorometholone eye drops. Rheumatology recommended completing a workup with an MRI angiogram of the brain with and without contrast and a biopsy before starting long-term immunosuppression. An MRI angiogram of the brain was obtained and was suspicious for a carotid-cavernous fistula (Figure 2). Neurointerventional radiology was consulted, and her antibiotics were de-escalated due to low suspicion for infectious etiology.

**Figure 3:**
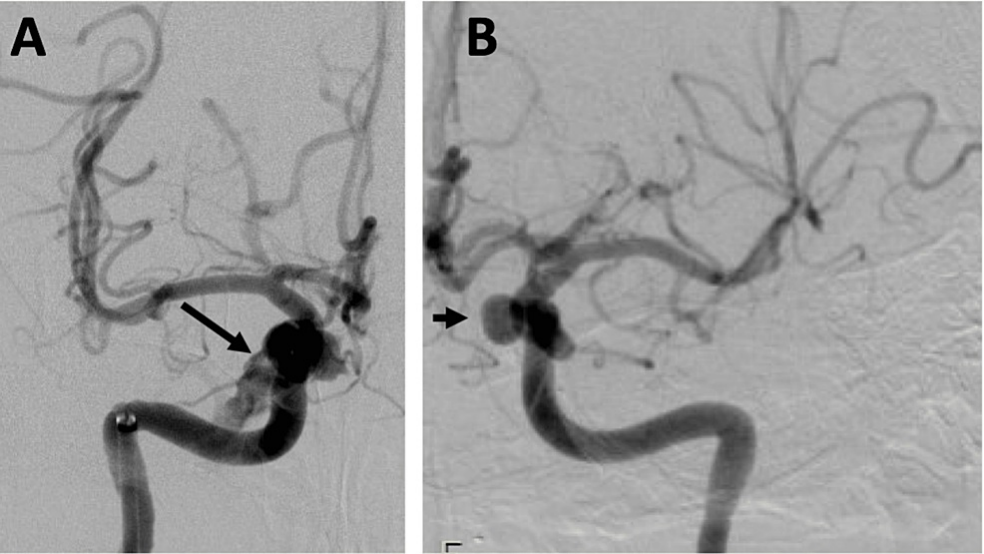
a: Cerebral angiogram showing a right-sided direct carotid-cavernous fistula (see long arrow) most likely secondary to a ruptured right internal carotid artery cavernous segment aneurysm. B: Cerebral angiogram showing a 6.5 mm saccular aneurysm of the cavernous segment of the left internal carotid artery (see short arrow).

The patient underwent a diagnostic cerebral angiogram and endovascular embolization with neuro-interventional radiology (Figure 3). She was diagnosed with a right-sided direct carotid-cavernous fistula, which was attributed to a ruptured right internal carotid artery cavernous segment aneurysm. It was during this procedure that she was also incidentally noted to have a 6.5 mm unruptured saccular aneurysm arising from the cavernous segment of her left internal carotid artery.

**Figure 4:**
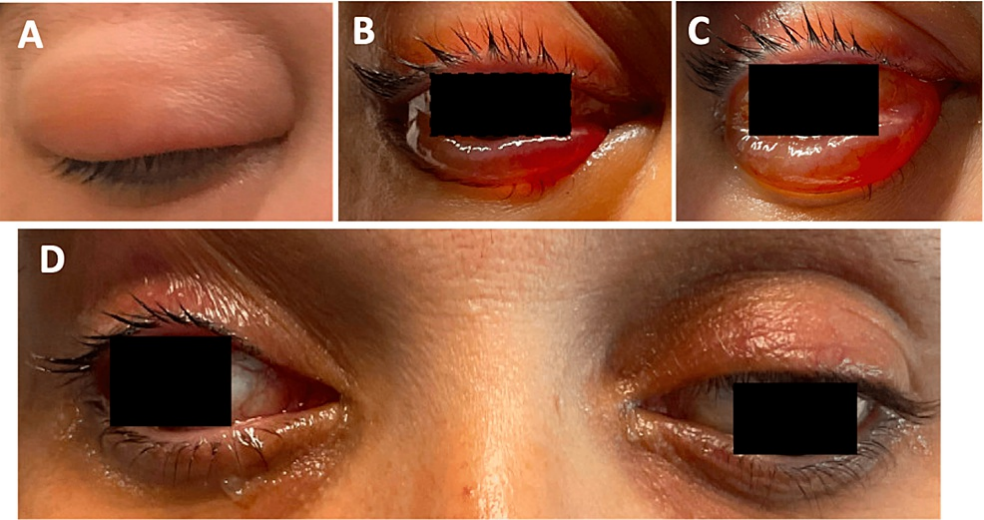
A) Day one: external view of the right eye with periorbital swelling and proptosis. B) Day two: right eye with chemosis (eye held open with assistance). C) Day three: right eye with continued chemosis (eye held open with assistance). D) Day seven: (post-op day one) improved chemosis and proptosis of the right eye with a continued right oculomotor nerve palsy (right eye held open with assistance).

In the following days, the patient experienced significant improvement in her periorbital edema and chemosis, although she did have a third cranial nerve palsy in the right eye (Figure 4). She was started on a short prednisone taper, and she no longer required pressure eyedrops for the right eye. The patient was discharged.

She followed up with ophthalmology outpatient in the following weeks. Eight weeks after discharge, her chemosis had resolved, and her diplopia and visual acuity had improved. Though she had a residual partial right oculomotor nerve palsy, her extraocular muscle movements were full.

Two months after the coil embolization of the right-sided carotid-cavernous fistula, the patient underwent successful coil embolization of the left internal carotid saccular aneurysm with planned neuro-interventional radiology follow-up.

## Discussion

The initial differential for this patient included rheumatologic conditions due to the presence of swelling around ocular structures, changes in the patient’s vision, and an initial response to steroid treatment. IgG4-related disease and idiopathic orbital inflammation were higher on the differential given the involvement of the orbit (a characteristically affected organ in this condition) and lab work-up that was negative for findings commonly seen in other autoimmune conditions, such as elevated erythrocyte sedimentation rate and C-reactive protein, and the autoantibodies that are associated with thyroid eye disease, myasthenia gravis, vasculitis, and lupus [2]. Sarcoidosis was also considered initially but was lower on the differential given the patient’s unremarkable chest X-ray; had the patient gotten a biopsy, the team would have further investigated this and the other rheumatologic conditions on the differential. Before a biopsy could be planned, however, radiologic imaging led to a vascular diagnosis that required intervention. Nevertheless, this case provided a useful opportunity to review the workup for a possible IgG4-related disease.

The criteria for diagnosing IgG4-related disease are complex, and while the patient’s IgG4 level was within normal limits, elevated IgG4 levels are neither sensitive nor specific for this diagnosis [1]. The clinical picture was initially unclear, as the lack of lacrimal gland inflammation suggested against IgG4-related disease, while the patient’s minimal response to steroids could have supported an autoimmune diagnosis. At the same time, the enlarged superior ophthalmic vein seen on CT imaging could have supported an underlying vascular phenomenon earlier in the diagnostic process.

Despite the initial breadth of possible diagnoses, the strict American College of Rheumatology and European League Against Rheumatism diagnostic criteria for IgG4-related disease include radiologic stipulations that ultimately led to the exclusion of this patient from this diagnosis and the correct identification of a carotid-cavernous fistula. According to these guidelines, rapid radiologic progression of symptoms and known radiologic findings suspicious for malignancy or infection are both sufficient alone to disqualify a patient from a diagnosis of IgG4-related disease [2]. While the patient in this case did not have serial imaging to document her symptom progression, the acute onset and severity of her proptosis in the absence of any indolent symptoms were suggestive of a non-rheumatologic cause. Likewise, while the guidelines do not include radiologic vascular abnormalities in the exclusion criteria, it was through following the guidelines and using radiology to rule out other potential diagnoses that this patient’s fistula was ultimately diagnosed.

Carotid-cavernous fistulas are abnormal connections between an artery and the cavernous sinus, and they are classified based on their etiology and the vessels involved [3]. According to this anatomical-angiographic classification system, this patient’s fistula was categorized as "spontaneous" because it was due to the probable rupture of a pre-existing aneurysm and "direct" because of the direct, high-flow connection between the main internal carotid artery and the cavernous sinus [3].

Because of the proximity of the cavernous sinus to the eye, there have been several documented cases where these patients initially presented with an ocular complaint, ranging in severity from a red eye to an orbital compartment syndrome requiring emergency canthotomy [4,5]. The increased vascular flow can also cause proptosis and nerve palsies [6]. While the presentation of this condition varies widely, direct fistulas necessitate prompt intervention due to their frequent association with visual disturbances, as seen in this patient, and their rapid progression [7]. While CT and MRI imaging may suggest signs of a fistula, the gold standard of diagnosis is a cerebral angiogram, and first-line treatment is typically transarterial or transvenous embolization [7]. The prognosis for a direct carotid-cavernous fistula after endovascular embolization is overall good, with low morbidity and virtually no mortality [6]. Given the encouraging prognosis of carotid-cavernous fistulas with prompt diagnosis and treatment, the possibility of this acute vascular abnormality should be considered whenever patients present with acute ocular complaints, like in this case.

## Conclusions

This patient’s case offers a valuable reminder for ophthalmologists and rheumatologists to remain aware of the possible conditions that may cause similar symptoms to those seen in IgG4-related diseases. Carotid-cavernous fistulas are key diagnoses that should not be missed in these patients with acute-onset periorbital and visual symptoms due to their potential for rapid progression and neurological impairment as well as their treatability. In this case, in the course of narrowing the differential, it was the rheumatologist consult and not the ophthalmologist consult who recommended a cerebral angiogram for work-up. Had mimickers of IgG4-related disease not been explored, this diagnosis may have been missed altogether. Thankfully, with proper adherence to diagnostic criteria for IgG4-related disease, it is possible to expeditiously differentiate between these two diagnoses while pursuing the proper work-up of autoimmune conditions.
